# Hypothesis: the subcellular senescence sequence of a mesophyll cell mirrors the cell origin and evolution

**DOI:** 10.1186/s43897-022-00048-7

**Published:** 2022-12-06

**Authors:** Su-Sheng Gan

**Affiliations:** Plant Biology Section, School of Integrative Plant Science, Cornell University, Ithaca, NY 14853 USA

## The outstanding question

The origin and evolution of modern cells have been not only a long sought scientific question but also an interesting topic to general public. Significant progress has been made towards the reconstruction of the hatcheries of the first cells by integrative analyses of geochemistry and phylogenomics of the inorganic ion environments of universal components of modern cells (Mulkidjanian et al. 2012). The mitochondria and chloroplasts are likely evolved from engulfed aerobic and photosynthetic prokaryotes that once lived as independent organisms and then formed respective endosymbiotic organelles (https://www.nature.com/scitable/ebooks/cell-biology-for-seminars-14760004/118242821/; (Lee and Hwang 2021). However, the origin and evolution of modern cells, especially the origin and evolution order of subcellular organelles remain elusive, mainly due to the lack of appropriate “fossil” record; evidence from fossils has been in strong support for organismal evolution.

## The hypothesis

**Fig. 1:**
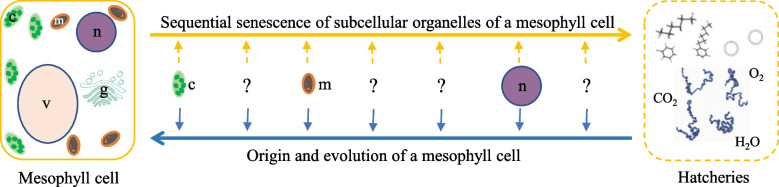
Schematic illustration of mirroring relationship between senescence progression and evolution of a plant mesophyll cell. The chloroplasts (c) are the first subcellular organelle to senesce and likely the last organelle to be evolved from endosymbiotic photosynthetic bacteria. The dismantlement of mitochondria (m) occurs at some time point after the chloroplast senescence, and the mitochondria is believed to be evolved by engulfing an aerobic bacterium prior to the formation of the chloroplasts. In contrast, the disintegration of the nucleus (n) is a relatively late event during senescence while the formation of the nucleus likely takes place early during cell evolution. The “soup” of the completely senescent cell resembles the ‘hatcheries” for the origin of the first protocell. Question marker (?) represents unknown event by which a subcellular organelle such as Golgi apparatus (g) or vacuole (v) is senescent or evolved. The arrowed yellow line indicates the order of subcellular senescence while the arrowed blue line represents the sequential processes of the cell origin and evolution

The active degenerative processes (generally referred to as senescence) of mesophyll cells may provide clues to the origin and evolution of the mesophyll cells. Here I present a hypothesis that the order of subcellular organelles senescence of a mesophyll cell mirrors the origin and evolution of the cell (Fig. 1). The first subcellular organelle to be dismantled (e.g., chloroplast) during senescence is the last subcellular organelle to be evolved, while the last subcellular organelle to be dissolved during senescence is likely the first subcellular organelle to be evolved. The “soup” of the completely senescent cells resembles the “hatcheries” of the very first cells (Fig. 1).

## Rationale/justification

Leaf mesophyll senescence is a genetically well programmed self-attrition process (Gan and Amasino 1997; Guo et al. 2021). Ecophysiologically, leaf senescence is not simply a degenerative process, but is also a recycling process in which nutrients released from senescing cells are translocated to actively growing organs or storage tissues. In many species, the pattern of leaf senescence illustrates this point: cells around the vascular system, which are required for nutrient export, are the last to senesce. In a natural setting, limited nutrients and water are major factors that threaten the plant life; senescence of old leaves (thus eliminating water consumption) and associated nutrient recycling are believed to contribute to the evolutionary fitness of plants (Gan and Amasino 1997). The subcellular and molecular biological events of a leaf cell senescence may well infer the above hypothesis regarding the origin and evolution of the mesophyll cells.

At the subcellular level there is a distinct pattern of mesophyll senescence. Several studies have shown that leaf mesophyll cells undergo defined subcellular changes during senescence. For example, the loss of chloroplast integrity occurs first, whereas the breakdown of nucleus is a relatively late event, and the breakdown of mitochondria takes place between the events (Inada et al. 1998; Cao et al. 2013; Zhou et al. 2019). Because a large portion of the nitrogen in a mesophyll cell is in the chloroplast, it is expected that during leaf senescence chloroplasts are broken down, while other cellular constituents such as the nucleus remain intact to accomplish cell attrition and the nutrient recycling process. The order of these subcellular senescence events mirrors the sequential events of chloroplast and mitochondria evolution. It has been proposed and generally accepted that the mitochondria and chloroplasts are likely evolved, respectively, from engulfed aerobic and photosynthetic bacteria that once lived as independent organisms, and that the chloroplasts evolved relatively lately and the formation of the mitochondria occurred prior to the chloroplast evolution event (e.g., https://www.nature.com/scitable/ebooks/cell-biology-for-seminars-14760004/118242821/).

At the molecular level, the senescence process is driven by a subset of senescence-associated genes (*SAG*s) that are expressed at the onset of and during senescence (Gan and Amasino 1997; Yuan et al. 2020; Guo et al. 2021; Cao et al. 2022; Wang et al. 2022). Some of the products of these *SAG*s are utilized to disassemble the subcellular organelles. It is thus reasonable to assume that those subcellular organelles that evolved the latest (such as chloroplasts) will be degraded first, and those machineries necessary for transcription and translation of *SAG*s will be dismantled last (relatively). The sequential events are consistent with the above hypothesis. It should be noted that the hypothesis does not imply that *SAG*s are among the first genes to be evolved during the origin and evolution of the cell.

## Approaches

Although there are some microscopic studies on the sequential processes of subcellular organelle senescence, many details are not known as shown in Fig. 1 with question markers. To address these questions, I propose to utilize, for example, different transgenic tobacco lines, in which individual subcellular organelles will be “tagged” with green fluorescence protein (GFP) or red fluorescence protein (RFP) (organelle specific protein-GFP or RFP fusion). Plants with different combination of the GFP-labeled and RFP-labeled subcellular components will be generated by various genetic crosses (for example, GFP-chloroplast x RFP-mitochondria, RFP-chloroplast x GFP-nucleus, GFP-chloroplast x RFP-tonoplast, or RFP-mitochondria x GFP-Golgi, etc.). The sequential disappearance of the GFP and RFP in each combination will be observed in senescing mesophyll cells of the tobacco leaf blade under confocal microscope. Senescence progresses from leaf tips to petiole. Thus, one leaf blade will have mesophyll cells at different senescence stages (from completely senescent cells at the blade tips to non-senescing cells close to the blade petiole). By doing so, we should be able to fine map the senescence order of the subcellular components in mesophyll cells.

## Conclusion

The origin and evolution of the plant cell is an intriguing yet important biological question that has been very difficult to tackle due to lack of “fossil” evidence. The hypothesis here represents a unique model for addressing the question. It also represents a novel aspect of leaf senescence in terms of evolution of leaf senescence.

## Data Availability

Not applicable.
